# Prospective memory impairment following whole brain radiotherapy in patients with metastatic brain cancer

**DOI:** 10.1002/cam4.1784

**Published:** 2018-09-27

**Authors:** Huaidong Cheng, Haijun Chen, Yue Lv, Zhendong Chen, Chiang‐Shan R. Li

**Affiliations:** ^1^ Department of Oncology The Second Affiliated Hospital of Anhui Medical University Hefei China; ^2^ Department of Psychiatry Yale University School of Medicine New Haven Connecticut

**Keywords:** brain metastases, prospective memory, radiotherapy

## Abstract

**Objective:**

To investigate the prospective memory (PM) impairment following whole brain radiotherapy (WBRT) in cancer patients with brain metastases.

**Method:**

Eighty‐one patients with metastatic brain cancer, agreeing to undergo WBRT, were enrolled and subjected to a battery of cognitive neuropsychological tests, including the mini‐mental state examination (MMSE), verbal fluency test (VFT), digit span test (DST), and event‐based and time‐based prospective memory (EBPM and TBPM) tasks, before and after radiotherapy.

**Results:**

The patients with metastatic brain cancer after WBRT exhibited a significant decrease in the MMSE, DST, VFT, and EBPM scores (*t* = 6.258, 10.192, 5.361, −5.892, *P *<* *0.01), but nonsignificant decrease in the TBPM scores (*t* = −1.172, *P *>* *0.05).

**Conclusion:**

There is significant EBPM impairment in cancer patients with brain metastases after WBRT, whereas that in the TBPM remained relatively unaffected. The result suggests that EBPM impairment may be as an early cognitive impairment marker in patients with BM who undergo WBRT.

## Abstract 2

There is significant EBPM impairment in cancer patients with brain metastases after WBRT, whereas that in the TBPM remained relatively unaffected. The result suggests that EBPM impairment may be as an early cognitive impairment marker in patients with BM who undergo WBRT.

## 1 INTRODUCTION

The morbidity of brain metastases (BM) increases significantly due to the extended survival of cancer patients, such that 20%‐40% of cancer patients exhibit brain metastases during the course of the disease.^1^ Overall, metastatic brain cancers have an unfavorable prognosis and a natural generation time of only one month,^2^ which means that the patients receiving therapy have a median survival period of 4 to 6 months.^3^ The widespread use of computed tomography and magnetic resonance imaging (MRI) in clinical practice has allowed the diagnosis of BM before the presentation of overt central nervous system symptoms. Currently, the treatment of BM includes whole brain radiotherapy (WBRT), stereotactic radiosurgery (SRS), surgical operation, chemotherapy, molecular targeting, and biotherapy,^4^ with WBRT being the most prominent treatment for patients with multiple BM. Malignant cells are particularly sensitive to X‐ray irradiation, and X‐ray therapy can be used to lyse malignant cells in the brain. However, X‐rays also cause nonspecific damage to healthy brain tissue, leading to cognitive impairment. Cognitive impairment associated with radiotherapy is defined as the changes in cognitive function during or after discontinuation of radiotherapy for cancer patients. For example, patients with small‐cell lung cancer (SCLC) and without brain tumors show a decline in cognitive function when receiving prophylactic cranial irradiation.^5^ A large number of studies have found that WBRT can significantly reduce and alleviate the symptoms of cancer patients, but these benefits may be outweighed by its adverse effects on neurocognitive function, such as the impairment of memory, executive function, and fine motor coordination, with the early changes being difficult to recognize.^6^

Memory is one of the most important cognitive functions and is closely related to the quality of life of cancer survivors. McDougall et al^7^ found that most cancer survivors exhibited varying degrees of memory impairment, which affected their return to the society. In cognitive neuropsychology, prospective memory (PM) is defined as the future plans or intentions of memory^8^ and is a memory component that is most closely related to daily activities. McDaniel et al^9^ classified PM into event‐based prospective memory (EBPM) and time‐based prospective memory (TBPM), which are required to perform a purposeful behavior in the presence of specific target events and goals.

However, it remains unknown whether prospective memory (including EBPM and TBPM) impairment is observed for cancer patients exhibiting BM, following radiotherapy. In the present study, we investigated whether there is a difference in EBPM and TBPM impairment in 81 cancer patients with BM, before and after WBRT by using the EBPM and TBPM neuropsychological tests.

## 2 MATERIALS AND METHODS

### 2.1 Participants

A total of 81 cancer patients with BM, who were hospitalized from January 2012 to December 2017 in the Department of Oncology, the Second Affiliated Hospital of Anhui Medical University, were recruited. Information regarding the patients’ age, education, and original lesion was gathered and statistically analyzed. Cancer patients with BM were selected based on the following criteria: (a) verification of the diagnosis of BM; (b) age ≥ 8 years; (c) willingness to participate in the study; (d) expected survival ≥ 3 months; (e) normal cognitive function, with a mini‐mental state examination (MMSE) score of ≥24; (f) ability to perform daily activities with a Karnofsky performance scale score of ≥80; and (g) no impairment in vision, hearing, and language. Additionally, cancer patients with BM were excluded based on the following criteria: (a) dementia; (b) clear effect on cognition due to a recent history of stroke; (c) psychiatric symptoms, such as anxiety and depression; (d) patients with cachexia or too weak to finish the test; (e) a history of alcohol and drug dependence; and (f) other factors such as serious adverse effects, chemotherapy and other treatment, serious edema which may lead to cognitive dysfunction and physical or mental illness. The study was approved by the Research Ethics Committee of the Second Affiliated Hospital of Anhui Medical University (Reference Number of Ethical Approval: 2 012088), and informed consent was obtained from all subjects.

### 2.2 WBRT

The WBRT involved the use of a linear accelerator, with an X‐ray of 6 MV. A hot plastic facial membrane was used to fix the head during localization and irradiation, and the MIMI machine was used to perform localization. The radiation field was determined using the horizontal cross section of the parallel sides, spanning the whole brain. Horizontal whole brain irradiation was performed, with the upper margin of the head, and from the lower and upper borders of the eyebrows to behind the orbit, down to the outer canthus, along the porus acusticus externus to the bottom of the posterior fossa, with a 2.0‐Gy dose each time, five times a week, resulting in a total dosage of 40 Gy over the course of treatment.

### 2.3 Neuropsychological background tests

According to the aforementioned grouping of cancer patients with BM, a series of neuropsychological background tests were administered within 1 week before radiotherapy, and 1 month after radiotherapy, to assess the cognitive functions, including memory. The MMSE was administered to assess the cognitive functions, including temporal and spatial orientation, short‐term memory, calculation, language, and visuo‐spatial skills.^10^ The verbal fluency test (VFT) was administered to the subjects, who were instructed to name as many animals as possible in one minute. The digit span test (DST) was used to measure short‐term memory, in which the subjects were instructed to recall a series of numbers after hearing them in a randomized order. The total score was determined by the number of digits recalled in the correct serial order. Similarly, the PM tasks were performed as follows.

### 2.4 EBPM task

Subjects were initially instructed to tap the desk whenever they found the two animal words (target events) during the task. They were requested to provide their telephone number after the tests were finished. Next, the subjects were given a word selection task, using 30 question cards. On each card, 12 Chinese words were printed. Ten of the 12 words belonged to one category, and the remaining two words belonged to another category. Subjects were instructed to select the two words that belonged to a category that differed from the other 10 words. The experimenter presented each card to the subjects, who were then instructed to answer verbally at their own pace. The target events for the PM task occurred on the 5th, 10th, 15th, 20th, 25th, and 30th cards of the word selection task. The subjects’ performance in the word selection task was recorded using a method similar to that reported by McDaniel et al One point was awarded for each correct response to a target event (total six target events). Two points were awarded for remembering to provide their telephone number after the test. No points were awarded for an incorrect response to a target event, or for forgetting to provide their telephone number. The maximum score in the EBPM task was 8.

### 2.5 TBPM task

Subjects were instructed to tap the desk at 5‐min intervals from the starting time (ie, at the time points of 5, 10, and 15 min). During the test, subjects were allowed to use a digital clock to check the time. To exclude any visible cues, the clock was placed one meter away, behind the subjects’ right shoulder, so that the subjects had to turn their head to check the time. The clock was set to display 0 hour 0 minute and 0 second at the beginning of the test. After the clock was started, the subjects were administered the number selection task, which included 100 cards. On each card, 12 two‐digit numbers were printed. Subjects were instructed to select the smallest and the largest numbers in the cards. The exact time at which the subjects responded by tapping the desk was recorded. The number selection task was stopped when the clock indicated 17 minutes. Two points were awarded if the subjects responded from 10 seconds before to 10 seconds after the target time. One point was awarded if the subjects responded from 30 seconds before to 30 seconds after the target time. The maximum score of the TBPM task was 6.

### 2.6 Evaluation of the effectiveness of radiotherapy for metastatic brain tumor

A repeat head MRI was performed 4 weeks after radiotherapy, according to the Response Evaluation Criteria for Solid Tumors version 1.1, to evaluate the effectiveness of radiotherapy against metastatic brain cancer.^11^ The Response Evaluation Criteria has several categories. The complete response occurs when there is a complete disappearance of all target lesions. The partial response (PR) occurs when there is at least a 30% decrease in the sum of the longest diameters (LDs) of target lesions, taking the baseline sum of LDs as the reference. Stable disease (SD) occurs when there is neither a sufficient shrinkage in the target lesion to qualify for PR nor a sufficient increase to qualify for progressive disease (PD), taking as reference the smallest sum of LDs at the time of treatment; PD occurs when there is at least a 20% increase in the sum of the LDs of target lesions, taking as reference the smallest sum of LDs recorded at the time of treatment or the appearance of one or more new lesions.

### 2.7 Statistical analysis

All data were expressed as mean ± standard deviation (SD). Statistical analysis was performed with SPSS software (version 22.0, http://spss.en.softonic.com/; Chicago, IL, USA). All data between before and after radiotherapy were analyzed by means of paired‐samples t tests. All statistical tests were two‐tailed, with the level of significance set at *P *<* *0.05.

## 3 RESULTS

### 3.1 The basic clinical information on patients with BM

A total of 81 patients (age: 58 ± 12 years) were enrolled, 49 symptomatic and 32 asymptomatic. A total of 59 patients had lung cancer; 18 had esophageal cancer, gastric cancer, and colon cancer; 1 had cervical cancer; and 3 had undefined types of cancer (Table 1).

**Table 1:** The basic clinical information of metastatic brain patients

Clinical characteristics	N
Sex	
Male	48
Female	33
Age (y)	
≥65	42
<65	39
Original lesion	
Lung cancer	49
Esophagus cancer, gastric cancer, and colon cancer	28
Cervical cancer	1
Undefined cancer	3
Metastatic lesion (n)	
≥3	27
<3	54
Max diameter of the metastatic brain lesion (cm)	
≥3	23
<3	58
Metastatic to other regions	
Yes	51
No	30
Symptoms of CNS^a^	
Yes	49
No	32

a In particular refers to symptoms caused by brain tissue damage or compressing, including dizzy, headache, sensory, motor dysfunction, and cognitive impairment, excluding symptoms caused by peripheral nerve or spinal cord damage.

### 3.2 Comparison of neuropsychological background tasks, EBPM, and TBPM following radiotherapy

According to the neuropsychological background tests results, the MMSE, DST, and VFT scores were significantly decreased (MMSE: 27.21 ± 1.27 vs 26.06 ± 1.05; DS: 7.70 ± 0.68 vs 6.46 ± 0.87; VFT: 10.01 ± 2.15 vs 8.37 ± 1.73; all *P *<* *0.01) after radiotherapy, and the EBPM scores of patients with BM were significantly decreased (2.48 ± 0.92 vs 1.54 ± 1.10; *P *<* *0.01); however, there is no statistical significance on TBPM scores between before and after radiotherapy (4.90 ± 1.02 vs 4.70 ± 1.12; *P *>* *0.05; Tables 2 and 3).

**Table 2:** Comparison of neuropsychological background testing between the two groups before and after radiotherapy

Group	N	DST	VFT	MMSE
Before radiotherapy	81	7.71 ± 0.68	10.02 ± 2.15	27.22 ± 1.28
After radiotherapy	81	6.46 ± 0.87^a^	8.37 ± 1.73^a^	26.07 ± 1.06^a^

DST, digit span test; MMSE, Mini‐mental state examination; VFT, verbal fluency test.

a *P *<* *0.01.

**Table 3:** Comparison of EBPM and TBPM scores between the two groups before and after radiotherapy

Group	N	EBPM	TBPM
Before radiotherapy	81	2.48 ± 0.92^a^	4.90 ± 1.10^b^
After radiotherapy	81	1.54 ± 1.10	4.70 ± 1.12

EBPM, event‐based prospective memory; TBPM, time‐based prospective memory.

a *P *<* *0.01.

b *P *>* *0.05.

### 3.3 Evaluation of the effectiveness of WBRT against BM

The effectiveness of WBRT in reducing tumor size for all patients was 95.1% (77/81) 1 month after treatment, including 34 PR cases and 43 SD cases. The success rate of WBRT in patients with central nervous system symptoms was 95.9% (47/49), with 40.8% (20/49) PR cases and 59.2% (29/49) SD cases. The success rate of WBRT in patients without central nervous system symptoms was 93.8% (30/32), with 31.2% (10/32) PR cases and 68.8% (22/32) SD cases.

## 4 DISCUSSION

The normal survival period for patients with metastatic brain cancer is about one to three months.^12^ Following WBRT, the median survival time is prolonged.^13^ Intracranial metastatic lesions can be controlled to a certain extent using WBRT, but it can also exert adverse effects on normal brain cells, leading to delayed, dose‐dependent neurological complications and cognitive abnormalities, thus, affecting the patients’ quality of life.^14^ However, the specific features of the cognitive impairment following WBRT in cancer patients with BM were unclear. In the past, because of the low survival rate of patients with BM, there have been few studies in this area. Recent studies have found that cognitive impairment due to WBRT is widespread; Chang et al^15^ found that the decline in cognitive function can be expressed as impaired learning and memory, and clinical manifestations ranging from mild cognitive dysfunction to severe dementia. Brummelman et al^16^ showed that radiotherapy‐related memory and executive function impairment may be associated with abnormal prefrontal and hippocampal function following radiotherapy and be associated with the total dose of radiotherapy.

In the current study, 81 patients with BM were administered the MMSE and PM tests (EBPM and TBPM) before and after WBRT. It was found that there was a decrease in the overall cognitive function following radiotherapy in patients with BM, and the EBPM scores were significantly lower (*P *<* *0.01). There was no statistically significant difference between the TBPM scores before and after radiotherapy, which provided direct evidence of EPPM impairment following WBRT in patients with BM.

Cognitive disorders involve different degrees of cognitive function impairment, varying from mild cognitive disorder to severe dementia.^17^ Patients with brain tumor commonly present symptoms of mild cognitive disorder, damaged memory, attention deficit, reduced illation and abstraction, and reduction of language skills.^18^ The cognitive impairment caused by WBRT involves changes in memory, implementation function, and information processing. The cognitive deficits in patients with BM with increased survival period have attracted increasing attention of physicians. It had been suggested that the major pathological changes in brain tissues following WBRT involve damage to vascular endothelial cells and demyelination of white matter.^19^ Based on the duration of the symptoms, there are three phases of radiological brain injury: acute, subacute, and late phase. During the acute phase, the major pathological changes are as follows: damage to vascular endothelial cells, increased capillary permeability, damage of blood‐brain barrier, and cerebral edema. The acute phase generally occurs within the first few weeks following radiotherapy and presents as fatigue, headache, and malignant vomiting. During the subacute, phase, the major changes are diffuse demyelination of white matter. It occurs 1‐6 months after radiotherapy, presenting as headache, somnolence, fatigue, and transient cognitive disorder. At the late phase, the major tissue changes are damage to vascular endothelial cells, demyelination of nerve fiber, and coagulation necrosis. This can occur 6 months after radiotherapy and presents as permanent and progressive memory loss, and even dementia in severe cases. At present, the hippocampus is considered to be one of the brain structures that is most closely related to cognitive function.^20^ Researchers have speculated that the cognitive disorder caused by WBRT may be associated with hippocampal damage. Patients with metastatic carcinoma involving the hippocampus were excluded from this study to minimize the proportion of cognitive disorder caused by direct hippocampal damage due to the tumor itself.

The specific mechanism underlying the cognitive impairment caused by WBRT is not clear and may be related to radiation‐induced brain tissue damage, brain edema following radiotherapy, free radical damage, functional connectivity changes, and other factors. There are many factors that affect a patient's cognitive function following WBRT, such as radiation dosage and segmentation. Tallet et al^21^ demonstrated that patients receiving a dosage less than 3 Gy had a significantly lower risk of cognitive dysfunction than those receiving a higher dosage. In this study, we used a regular dosage for WBRT, with a total dosage of 40 Gy. It is worth noting that Aoyama et al^6^ showed that controlling the BM is the most significant factor in stabilizing cognitive function. Another prospective study, conducted at the University of Wisconsin, analyzed the influence of WBRT on cognitive function in patients with BM.^22^ The authors found that WBRT had shrunken the tumors, improved patients’ survival period, and rescued their cognitive function. The long‐term survivors exhibited stable and improved cognitive function. Moreover, the cognitive impairment due to tumor growth was much larger than that due to WBRT. Besides the effect of WBRT on cognitive function, there are other factors that affect the cognition of the patients, such as age,^23^ complications,^24^ tumor location,^25^ epilepsy^26^ due to the tumor, surgery,^27^ certain drugs,^28^ chemotherapy,^29^ the controlling situation of intracranial or extracranial diseases, as well as neurological diseases.

This study on cerebral irradiation and cognition is important because it could provide information on how to prevent and reduce the cognitive damage that occurs during the treatment of metastatic brain cancer. This is extremely important for cancer patients with long‐term survival because cognitive impairment may diminish the life quality of such patients. When performing cerebral irradiation, it is important to avoid key brain regions (such as the hippocampus), which have a small probability of being the sites of metastasis but damaging which could significantly impair the cognitive function of the patient.^20^, ^30^, ^31^ In such cases, intensity‐modulated radiotherapy and helical tomotherapy may provide the information necessary to avoid complications.^32^, ^33^ Further, drugs such as memantine could help prevent or ameliorate the cognitive impairment induced by irradiation.^34^

Adding to the previous studies on memory impairment following WBRT, in this study, we found that patients with BM exhibited significantly lower EBPM scores. Impairment of PM is the most common type of memory impairment, accounting for 50%‐80% cases. Previous studies have found that PM can be divided into at least two parts: EBPM and TBPM, through the establishment of cognitive neural psychology dual‐channel processing model. Many studies have found that the two components of PM can be separated, with different neural mechanisms underlying the brain damage, supported by neuroimaging evidences. The EBPM task is more relevant to assess prefrontal cortical function,^35^ whereas Oksanen et al^36^ did not find the activation of the prefrontal lobe to be associated with TBPM tasks, which may be associated with other structures, such as the thalamus.^37^ This study found that the EBPM score is significantly lower in patients with BM, following WBRT, and may be associated with a decrease in frontal lobe function. Therefore, WBRT may lead to decline in frontal lobe function in patients with BM. In addition, the functional brain imaging study of patients with BM before and after WBRT was further elucidated the possible mechanism underlying lower EBPM scores following WBRT. The result is the first time to found the EBPM impairment caused by WBRT in cancer patients with brain metastases, but there is a limitation to consider. Because the survival period of enrolled patients was relatively short, the data on long‐term cognitive changes following WBRT are not available in our study.

## 5 CONCLUSION

In conclusion, present study demonstrates that patients with BM who undergo WBRT exhibit impaired cognition, performing poorly in the EBPM task, but with little impact on performance in the TBPM task. The result indicated that WBRT exerts heterogeneous effects on PM, and EBPM impairment may be recognized as an early behavior marker in patients with BM who undergo WBRT.

## CONFLICT OF INTEREST

The authors declare no conflict of interest, and the publication of this manuscript was approved by all authors.

## References

[1] Tsao MN , Lloyd N , Wong RK , et al. Whole brain radiotherapy for the treatment of newly diagnosed multiple brain metastases. Cochrane Database Syst Rev. 2012;(4):CD003869. 22513917 10.1002/14651858.CD003869.pub3 PMC6457607

[2] Renfrow JJ , Lesser GJ . Molecular subtyping of brain metastases and implications for therapy. Curr Treat Options Oncol. 2013;14(4):514‐527. 23907440 10.1007/s11864-013-0248-2

[3] Buglione M , Pedretti S , Gipponi S , et al. The treatment of patients with 1‐3 brain metastases: is there a place for whole brain radiotherapy alone, yet? A retrospective analysis Radiol Med. 2015;120(12):1146‐52. 25917339 10.1007/s11547-015-0542-0

[4] Habets EJ , Dirven L , Wiggenraad RG , et al. Neurocognitive functioning and health‐related quality of life in patients treated with stereotactic radiotherapy for brain metastases: a prospective study. Neuro‐oncology. 2016;18(3):435‐444. 26385615 10.1093/neuonc/nov186 PMC4767242

[5] Sun A , Bae K , Gore EM , et al. Phase III trial of prophylactic cranial irradiation compared with observation in patients with locally advanced non‐small‐cell lung cancer: neurocognitive and quality‐of‐life analysis. J Clin Oncol. 2011;29(3):279‐286. 21135267 10.1200/JCO.2010.29.6053 PMC3056463

[6] Aoyama H , Tago M , Kato N , et al. Neurocognitive function of patients with brain metastasis who received either whole brain radiotherapy plus stereotactic radiosurgery or radiosurgery alone. Int J Radiat Oncol Biol Phys. 2007;68(5):1388‐1395. 17674975 10.1016/j.ijrobp.2007.03.048

[7] McDougall GJ Jr , Oliver JS , Scogin F . Memory and cancer: a review of the literature. Arch Psychiatr Nurs. 2014;28(3):180‐186. 24856270 10.1016/j.apnu.2013.12.005 PMC4033831

[8] Arnold NR , Bayen UJ , Bohm MF . Is prospective memory related to depression and anxiety? A hierarchical MPT modelling approach. Memory. 2015;23(8):1215‐1228. 25337864 10.1080/09658211.2014.969276

[9] McDaniel MA , Einstein GO . The neuropsychology of prospective memory in normal aging: a componential approach. Neuropsychologia. 2011;49(8):2147‐2155. 21192957 10.1016/j.neuropsychologia.2010.12.029 PMC3095717

[10] Shibamoto Y , Baba F , Oda K , et al. Incidence of brain atrophy and decline in mini‐mental state examination score after whole‐brain radiotherapy in patients with brain metastases: a prospective study. Int J Radiat Oncol Biol Phys. 2008;72(4):1168‐1173. 18495375 10.1016/j.ijrobp.2008.02.054

[11] Eisenhauer EA , Therasse P , Bogaerts J , et al. New response evaluation criteria in solid tumours: revised RECIST guideline (version 1.1). Eur J Cancer. 2009;45(2):228‐247. 19097774 10.1016/j.ejca.2008.10.026

[12] Claus EB . Neurosurgical management of metastases in the central nervous system. Nat Rev Clin Oncol. 2011;9(2):79‐86. 22143137 10.1038/nrclinonc.2011.179

[13] Komatsu T , Kunieda E , Oizumi Y , Tamai Y , Akiba T . Clinical characteristics of brain metastases from lung cancer according to histological type: pretreatment evaluation and survival following whole‐brain radiotherapy. Mol Clin Oncol. 2013;1(4):692‐698. 24649230 10.3892/mco.2013.130 PMC3915483

[14] Marsh JC , Gielda BT , Herskovic AM , Abrams RA . Cognitive sparing during the administration of whole brain radiotherapy and prophylactic cranial irradiation: current concepts and approaches. J Oncol. 2010;2010:198208. 20671962 10.1155/2010/198208 PMC2910483

[15] Chang EL , Wefel JS , Hess KR , et al. Neurocognition in patients with brain metastases treated with radiosurgery or radiosurgery plus whole‐brain irradiation: a randomised controlled trial. Lancet Oncol. 2009;10(11):1037‐1044. 19801201 10.1016/S1470-2045(09)70263-3

[16] Brummelman P , Sattler MG , Meiners LC , et al. Cognitive performance after postoperative pituitary radiotherapy: a dosimetric study of the hippocampus and the prefrontal cortex. Eur J Endocrinol. 2012;166(2):171‐179. 22071311 10.1530/EJE-11-0749

[17] Arnsten AF , Wang M . Targeting prefrontal cortical systems for drug development: potential therapies for cognitive disorders. Annu Rev Pharmacol Toxicol. 2016;56:339‐360. 26738476 10.1146/annurev-pharmtox-010715-103617 PMC4734124

[18] Wu X , Gu M , Zhou G , Xu X , Wu M , Huang H . Cognitive and neuropsychiatric impairment in cerebral radionecrosis patients after radiotherapy of nasopharyngeal carcinoma. BMC Neurol. 2014;14:10. 24418214 10.1186/1471-2377-14-10 PMC3897961

[19] Yoo DH , Song SW , Yun TJ , et al. MR imaging evaluation of intracerebral hemorrhages and T2 hyperintense white matter lesions appearing after radiation therapy in adult patients with primary brain tumors. PLoS ONE. 2015;10(8):e0136795. 26322780 10.1371/journal.pone.0136795 PMC4556481

[20] Mattfeld AT , Stark CE . Functional contributions and interactions between the human hippocampus and subregions of the striatum during arbitrary associative learning and memory. Hippocampus. 2015;25(8):900‐911. 25560298 10.1002/hipo.22411 PMC4492918

[21] Tallet AV , Azria D , Barlesi F , et al. Neurocognitive function impairment after whole brain radiotherapy for brain metastases: actual assessment. Radiat Oncol. 2012;7:77. 22640600 10.1186/1748-717X-7-77 PMC3403847

[22] Li J , Bentzen SM , Renschler M , Mehta MP . Regression after whole‐brain radiation therapy for brain metastases correlates with survival and improved neurocognitive function. J Clin Oncol. 2007;25(10):1260‐1266. 17401015 10.1200/JCO.2006.09.2536

[23] Metzler‐Baddeley C , Jones DK , Steventon J , Westacott L , Aggleton JP , O'Sullivan MJ . Cingulum microstructure predicts cognitive control in older age and mild cognitive impairment. J Neurosci. 2012;32(49):17612‐17619. 23223284 10.1523/JNEUROSCI.3299-12.2012 PMC6621654

[24] Corn BW , Moughan J , Knisely JP , et al. Prospective evaluation of quality of life and neurocognitive effects in patients with multiple brain metastases receiving whole‐brain radiotherapy with or without thalidomide on Radiation Therapy Oncology Group (RTOG) trial 0118. Int J Radiat Oncol Biol Phys. 2008;71(1):71‐8. 18164829 10.1016/j.ijrobp.2007.09.015

[25] Klein M , Heimans JJ , Aaronson NK , et al. Effect of radiotherapy and other treatment‐related factors on mid‐term to long‐term cognitive sequelae in low‐grade gliomas: a comparative study. Lancet. 2002;360(9343):1361‐1368. 12423981 10.1016/s0140-6736(02)11398-5

[26] Iuvone L , Peruzzi L , Colosimo C , et al. Pretreatment neuropsychological deficits in children with brain tumors. Neuro‐oncology. 2011;13(5):517‐524. 21372071 10.1093/neuonc/nor013 PMC3093333

[27] An LN , Yue Y , Guo WZ , et al. Surgical trauma induces iron accumulation and oxidative stress in a rodent model of postoperative cognitive dysfunction. Biol Trace Elem Res. 2013;151(2):277‐283. 23229539 10.1007/s12011-012-9564-9

[28] Douw L , Klein M , Fagel SS , et al. Cognitive and radiological effects of radiotherapy in patients with low‐grade glioma: long‐term follow‐up. Lancet Neurol. 2009;8(9):810‐818. 19665931 10.1016/S1474-4422(09)70204-2

[29] Anderson NE , Posner JB , Sidtis JJ , et al. The metabolic anatomy of paraneoplastic cerebellar degeneration. Ann Neurol. 1988;23(6):533‐540. 3261571 10.1002/ana.410230602

[30] Marsh JC , Herskovic AM , Gielda BT , et al. Intracranial metastatic disease spares the limbic circuit: a review of 697 metastatic lesions in 107 patients. Int J Radiat Oncol Biol Phys. 2010;76(2):504‐512. 20117288 10.1016/j.ijrobp.2009.02.038

[31] Regine WF , Schmitt FA , Scott CB , et al. Feasibility of neurocognitive outcome evaluations in patients with brain metastases in a multi‐institutional cooperative group setting: results of Radiation Therapy Oncology Group trial BR‐0018. Int J Radiat Oncol Biol Phys. 2004;58(5):1346‐52. 15050309 10.1016/j.ijrobp.2003.09.023

[32] Gondi V , Tome WA , Marsh J , et al. Estimated risk of perihippocampal disease progression after hippocampal avoidance during whole‐brain radiotherapy: safety profile for RTOG 0933. Radiother Oncol. 2010;95(3):327‐331. 20392503 10.1016/j.radonc.2010.02.030 PMC2981132

[33] Bender ET , Mehta MP , Tome WA . On the estimation of the location of the hippocampus in the context of hippocampal avoidance whole brain radiotherapy treatment planning. Technol Cancer Res Treat. 2009;8(6):425‐432. 19925026 10.1177/153303460900800604 PMC2797122

[34] Brown PD , Pugh S , Laack NN , et al. Memantine for the prevention of cognitive dysfunction in patients receiving whole‐brain radiotherapy: a randomized, double‐blind, placebo‐controlled trial. Neuro‐oncology. 2013;15(10):1429‐37. 23956241 10.1093/neuonc/not114 PMC3779047

[35] Cheng HD , Wang K , Xi CH , Niu CS , Fu XM . Prefrontal cortex involvement in the event‐based prospective memory: evidence from patients with lesions in the prefrontal cortex. Brain Inj. 2008;22(9):697‐704. 18608204 10.1080/02699050802263035

[36] Oksanen KM , Waldum ER , McDaniel MA , Braver TS . Neural mechanisms of time‐based prospective memory: evidence for transient monitoring. PLoS ONE. 2014;9(3):e92123. 24643226 10.1371/journal.pone.0092123 PMC3958452

[37] Cheng H , Tian Y , Hu P , Wang J , Wang K . Time‐based prospective memory impairment in patients with thalamic stroke. Behav Neurosci. 2010;124(1):152‐158. 20141290 10.1037/a0018306

